# Identification of P450 Candidates Associated with the Biosynthesis of Physalin-Class Compounds in *Physalis angulata*

**DOI:** 10.3390/ijms241814077

**Published:** 2023-09-14

**Authors:** Congkun Hua, Zhengqin Xu, Nan Tang, Yehan Xu, Yansheng Zhang, Changfu Li

**Affiliations:** Shanghai Key Laboratory of Bio-Energy Crops, Research Center for Natural Products, Plant Science Center, School of Life Sciences, Shanghai University, Shanghai 200444, China; huacongkun@shu.edu.cn (C.H.); adrianxu021@126.com (Z.X.); nantang@shu.edu.cn (N.T.); xuyehan@shu.edu.cn (Y.X.); zhangys1@shu.edu.cn (Y.Z.)

**Keywords:** *Physalis angulata*, physalin, full-length transcriptome, P450

## Abstract

The *Physalis* genus has long been used as traditional medicine in the treatment of various diseases. Physalins, the characteristic class of compounds in this genus, are major bioactive constituents. To date, the biogenesis of physalins remains largely unknown, except for the recently established knowledge that 24-methyldesmosterol is a precursor of physalin. To identify the genes encoding P450s that are putatively involved in converting 24-methyldesmosterol to physalins, a total of 306 P450-encoding unigenes were retrieved from our recently constructed *P. angulata* transcriptome. Extensive phylogenetic analysis proposed 21 P450s that might participate in physalin biosynthesis. To validate the candidates, we developed a virus-induced gene silencing (VIGS) system for *P. angulata*, and four P450 candidates were selected for the VIGS experiments. The reduction in the transcripts of the four P450 candidates by VIGS all led to decreased levels of physalin-class compounds in the *P. angulata* leaves. Thus, this study provides a number of P450 candidates that are likely associated with the biosynthesis of physalin-class compounds, forming a strong basis to reveal the unknown physalin biosynthetic pathway in the future.

## 1. Introduction

**Figure 1:**
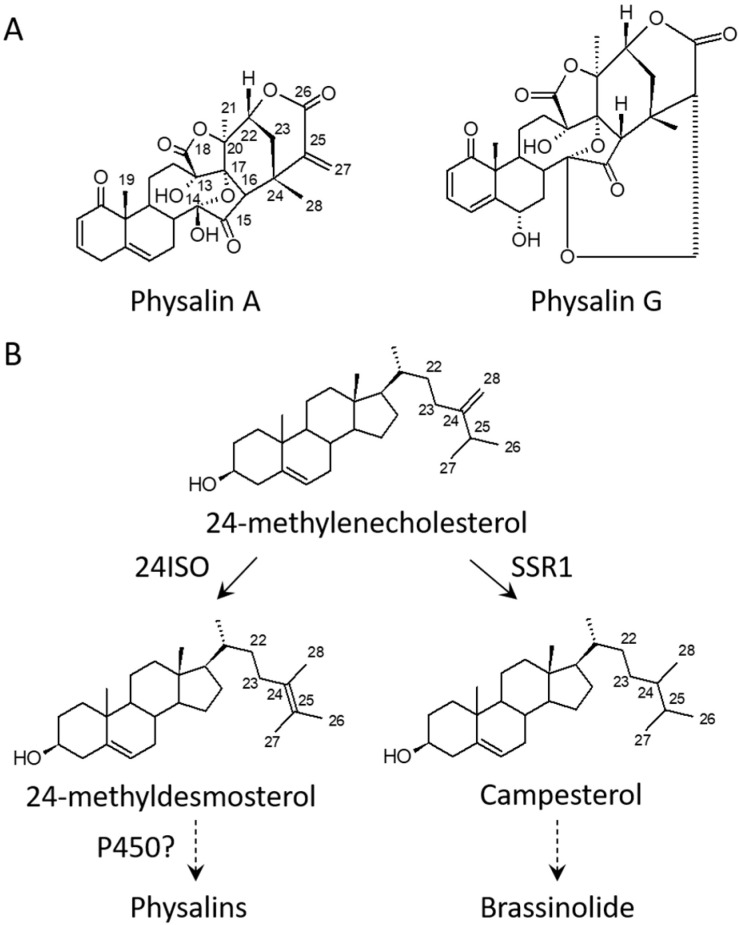
Proposed pathway for the biosynthesis of physalins in *Physalis angulata* starting from 24-methylenecholesterol. (**A**), Representative structures of physalins exampled by physalin A and physalin G; (**B**), Proposed pathway to physalins from the intermediate 24-methylenecholesterol. The 24-methylenecholesterol represents a branching point either toward biosynthesis of physalin via 24-methyldesmosterol under the action of 24ISO or to biosynthesis of brassinolide via campesterol catalyzed by SSR1 [8,9]. 24ISO, a sterol delta-24-isomerase; SSR1, a sterol side chain reductase 1; Solid line denotes one-step reaction, and dotted line means multi-step reactions.

*Physalis angulata*, commonly called Ku-Zhi in Chinese, belongs to the family *Solanaceae*. As a folk medicine mainly used in China, and Southeast Asian countries, this species has long been used in the treatment of malaria, dermatitis, tracheitis, arthritis, and hepatitis [1]. The main biological compounds in *P. angulata* are physalins, which have an ergostane-type skeleton with a lactone group formed between C-22 and C-26 [2]. Physalins appear only in the *physalis* genus, and they differentiate from other ergostane-type steroids by the cleavage of C13–C14 bonds and the formation of C16–C24 carbocyclic bonds (Figure 1A) [3]. A variety of pharmacological activities, such as the antinociceptive properties of physalin G [4], inhibition of breast cancer by physalin B [5], and prevention of bone loss by physalin D [6], was displayed by physalins.

Despite the medicinal importance of physalins, the pathway for physalins is largely unknown. Physalin forms a major subclass of withanolide [2], and 24-methylenecholesterol is an intermediate in the biosynthesis of withanolide [7]. In plants, the pathway leading to 24-methylenecholesterol has been elucidated [8]. The 24-methylenecholesterol represents a metabolic divergence point, from which the carbon flux is directed either towards biosynthesis of brassinolide via campsterol [8] or into biosynthesis of withanolide via 24-methyldesmosterol (Figure 1B) [9]. The conversion of 24-methylenecholesterol to 24-methyldesmosterol is catalyzed by a sterol delta-24-isomerase (24ISO) [9]. The 24ISO gene was recently cloned from *P. angulata* and its involvement in physalin biosynthesis was demonstrated by suppressing its expression in *P. angulata* [10]. In contrast, at present, enzymes involved in converting 24-methyldesmosterol to physalins have not been determined. Based on their chemical structures, physalin is produced from 24-methyldesmosterol through a series of oxidations on several carbon positions (Figure 1B), implying that diverse P450 enzymes are involved in these latter steps. Third-generation sequencing provides accurate full-length transcripts, and in recent years, it has accelerated the identification of genes involved in the biosynthesis of plant secondary metabolites. Recently, we applied this up to-date technique to the pooled mRNA samples extracted from the roots, stems, leaves and calyxes P. angulata [11], revealing the full-length transcripts of this species.

In this study, to identify putative P450s involved in physalin biosynthesis, we retrieved all the P450 candidates from this full-length transcriptome of *P. angulata,* and subjected them to an extensive phylogenetic analysis with previously known P450s involved in different metabolic pathways. The phylogenetic analysis proposed a number of P450 candidates that were possibly involved in the biosynthesis of physalins. To validate involvement of the P450 candidates in physalin biosynthesis, an efficient virus-induced gene silencing (VIGS) system for *P. angulata* was established in this study. When four P450s among the candidates were selected for the VIGS experiments, the silencing of their transcripts all led to a dramatic decrease in the biosynthesis of physalin H and dihydrophysalin A in the *P. angulata* leaves. Thus, the P450 candidates identified by this study would be valuable for further understanding of the pathway beyond 24-methyldesmosterol in physalin biosynthesis.

## 2. Results

### 2.1. Confirmation of the VIGS System for P. angulata

**Figure 2:**
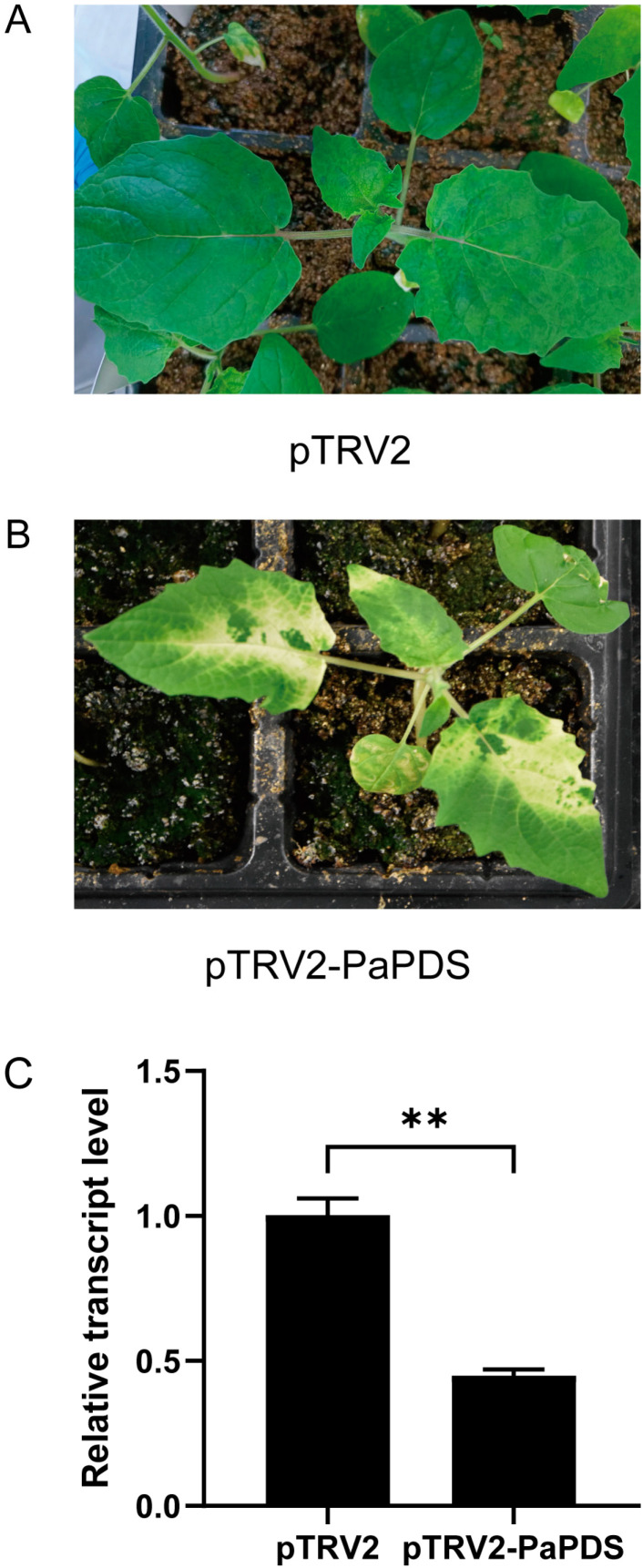
Silencing of *PaPDS* in *Physalis angulata* by VIGS. Representative phenotype of newly grown leaves of the infected control (**A**) and the *PaPDS*-silenced (**B**) *P. angulata* plants at around 14 days post-infection. (**C**) Real-time PCR analysis of the *PaPDS* transcript in the *PaPDS*-silenced leaves in comparisons with the controls. Difference was considered highly significant when ** *p* < 0.01.

The reporter gene *PaPDS* (Figure S1) was cloned from the *P. angulata* transcriptome. Approximately 254 bp-fragment of *PaPDS* was amplified as a target sequence for silencing, and ligated into the pTRV2 vector to yield the plasmid pTRV2-PaPDS. The pTRV1 and pTRV2-PaPDS were separately transformed into the *Agrobacterium* GV3101. The transgenic *Agrobacterium* culture containing the pTRV1 and pTRV2-PaPDS was infiltrated into the *P. angulata* leaves. As a control, the *Agrobacterium* fluid bearing the empty vectors of pTRV1 and pTRV2 was infiltrated. After two weeks post infiltration, a greenish-whitish variegated phenotype occurred in the newly emerging leaves of the pTRV2-PDS transformed plants (Figure 2B) but not in the controls (Figure 2A). We observed no visible photobleaching phenotype in the stems.

The white phenotype indicated reduced levels of *PaPDS* in the infected leaves by VIGS. We measured the *PaPDS* transcript levels in the *PaPDS*-silenced leaves in comparison with the controls by real-time PCR analysis. As expected, the transcript level of *PaPDS* was substantially knocked down in the *PaPDS*-silenced leaves compared with the corresponding controls (Figure 2C). This data clearly suggests that the VIGS is effective for knocking down the expression of genes in the *P. angulata* leaves.

### 2.2. Identification of the P450 Candidates Associated with the Biosynthesis of Physalin-Class Compounds

**Figure 3:**
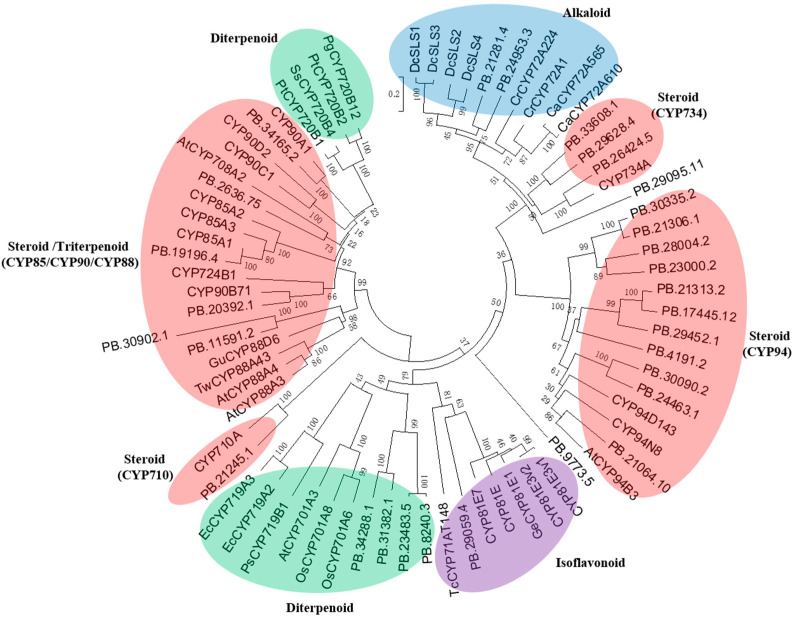
Phylogenetic analysis of the 21 *Physalis angulata* P450 candidates with the previously published P450s that are known to participate in biosynthesis of different types of secondary metabolites. Sequences were aligned by ClustalW [13]. The tree was constructed by the neighbor-joining method using MEGA software (MEGA 6.0) with 1000 bootstrap replicates. Accession numbers of the previously characterized P450s are shown in Table S1.

The pathway from 24-methyldesmosterol to physalins remains largely unknown, and by judging their molecular structures, we suggested possible involvement of various oxidations in physalin biosynthesis. Given that P450s usually catalyze the oxidation reaction in plant secondary metabolism [12], we sought to identify P450-encoding unigenes from the *P. angulata* transcriptome. The *P. angulata* transcriptome was searched, resulting in 306 sequences annotated as P450s. Among them, 73 sequences showed significant expression levels (FPKM value > 1.0). These 73 P450s were then subjected to phylogenetic analysis with various previously published P450s with known roles in biosynthesis of different types of plant secondary metabolites (see their accession numbers in Table S1). There were 21 P450s that showed a closer relationship to the triterpenoid/ steroid metabolism (Figure 3). Because physalins belong to steroid-class compounds which share a common biosynthetic pathway until the intermediate 2,3-oxidosqualene with triterpenoids, the identified 21 P450s (see their predicted amino acid sequences in Table S2), which were related to steroid or triterpenoid metabolism, constitute the candidates that are worthy of being further investigated for their possible roles in physalin biosynthesis.

**Figure 4:**
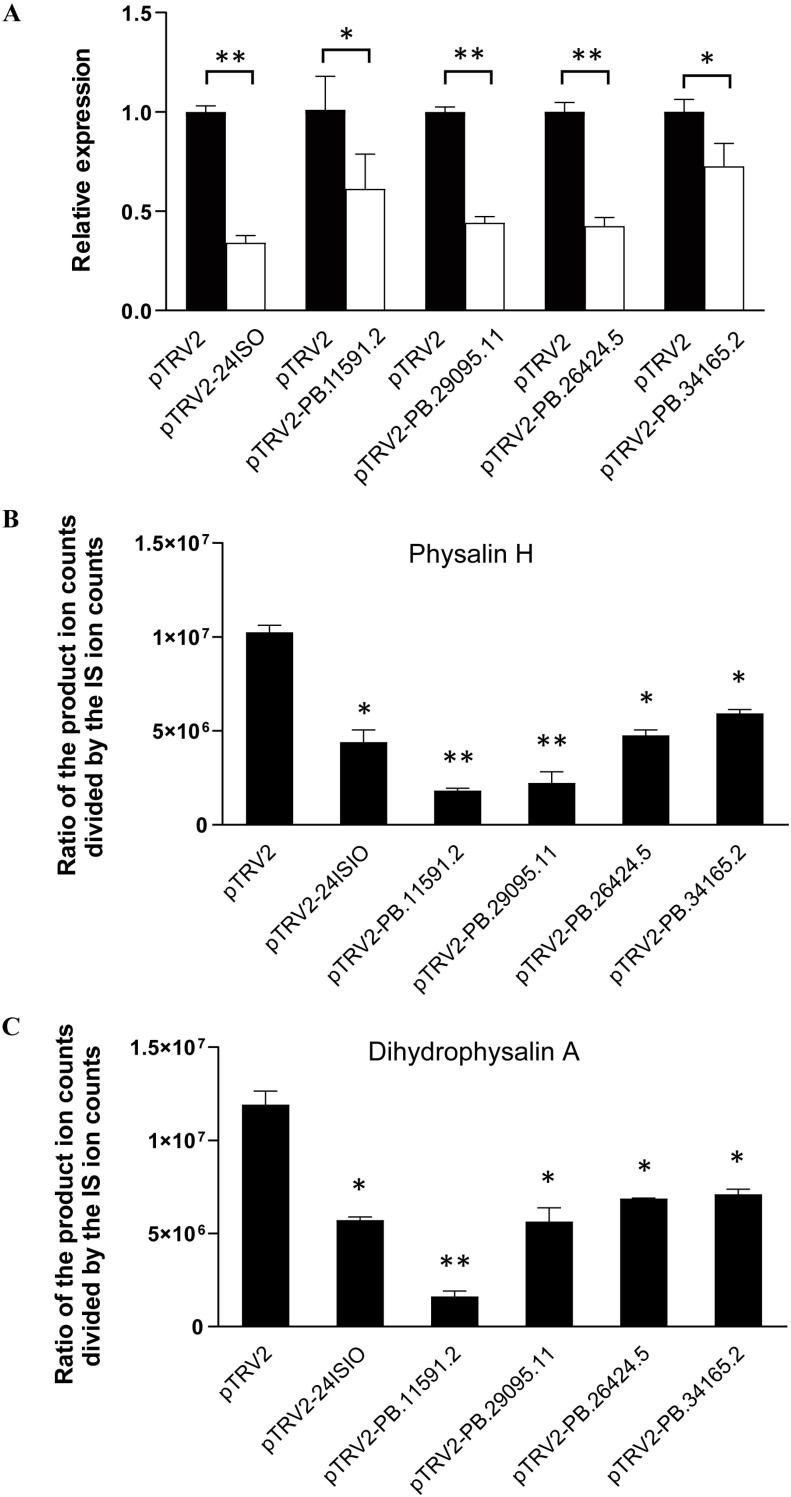
In planta silencing of the P450 candidates led to reduction in physalin biosynthesis in *Physalis angulata*. The 24ISO and the four P450 candidates (PB.34165.2, PB.26424.5, PB.11591.2, and PB.29095.11) were separately subjected to the VIGS experiment. (**A**), Transcript abundance of the targeted genes in their respective silenced leaves compared to the empty vector-transferred control; (**B**), The content of physalin H in the silenced *P. angulata* leaves compared with the empty vector control; (**C**), The content of dihydrophysalin A in the silenced *P. angulata* leaves compared with the empty vector control. Difference was considered statistically significant when * *p* < 0.05 and highly significant when ** *p* < 0.01.

To validate the P450 candidates identified by this study, we conducted experiments to down-regulate their expressions in vivo by VIGS. Considering that four P450 candidates (PB.34165.2, PB.26424.5, PB.11591.2 and PB.29095.11) showed a comparable expression level with the upstream gene *24ISO* in the pathway (Figure 1B), they were selected for the silencing experiment. Four-week-old plants were separately infiltrated with agrobacterium strains containing each of the P450 VIGS constructs, and the empty vector-transformed plants served as negative controls. The *24ISO*-silenced leaves were also prepared as a positive control. After two weeks post the infection, newly developed leaves were harvested to evaluate the silencing effects. Real-time PCRs confirmed that the transcript levels of the *24ISO* and the four P450 candidates were 27.36–65.83% down-regulated in the corresponding silenced plants, compared to the empty vector-transferred controls (Figure 4A). The silenced leaves were then subjected to LC-MS/MS analysis for the detection of physalins. Initially, the leaf extracts were measured by liquid chromatography-mass spectrometry (LC-MS) using targeted analysis for physalin standards (physalin A, physalin G, physalin D, physalin L, and isophysalin A). We did not find the peaks that exactly showed the same retention times and mass fragmentation patterns as these standards, however, based on the ion fragmentation behaviors for physalins summarized in previous literatures [14,15], abundant physalin-class compounds were apparently detected in the extracts. Huang et al. identified a number of diagnostic ions, such as product ions at *m*/*z* 121, 123, 133, 149, 151, 153, 157, 165, 173, 181, and 193, for different types of physalins [15], and these diagnostic ions were successfully used to classify the types of physalins [14]. Two major physalins (see their mass fragmentation patterns in Figures S2 and S3), identified as physalin H and dihydrophysalin A by comparing their MS data with the previous literature [14,15], were found in the leaf extracts. On comparison with the vector control leaves, the contents of physalin H and dihydrophysalin A were decreased by 57% and 52%, respectively in the *24ISO*-silenced plants (Figure 4B,C). With respect to the controls, a significant decrease in the content of both physalin H and dihydrophysalin A in the P450s-silenced leaves was recorded with the decrease percentage being 40–86% (Figure 4B,C).

## 3. Discussion

Some of physalins are considered as promising anti-tumor drug candidates due to their significant inhibition against several cancer cells but not regular human cells [16]. The contents of physalins are very low in plants, and many isoforms of them co-exist in nature, making it very challenging to separate them from plants in an industrial scale. Microbial engineering technique provides an alternative avenue to produce specific physalins by inserting their biosynthetic genes into the genome of microorganisms. However, the prerequisite of this up-to-date technique is to understand the biosynthetic steps toward specific physalins. A previous study suggested the role of 24-methyldesmosterol as a key precursor for the biosynthesis of withanolide to which physalin belongs [9]. Consistent with this finding, we recently confirmed a role of 24ISO in physalin biosynthesis in *P. angulata* by suppressing its expression in vivo [10]. Currently, further steps beyond 24-methyldesmosterol for physalin biosynthesis are completely unknown, and presumably a number of P450s contribute to a series of oxidizations in the pathways. Thus, this study focused on identifying possible P450 candidates involved in physalin biosynthesis.

A total of 306 sequences annotated as P450s were found in the *P. angulata* transcriptome, of which 73 transcripts showed a significant expression level (FPKM value > 1.0) in the sequenced tissues. We performed extensive phylogenetic analysis of the 73 *P. angulata* P450s, together with other P450s with known functions in biosynthesis of different types of plant secondary metabolites. The phylogenetic data showed the highest number of P450s that were associated with steroid biosynthesis, consistent with the fact that phytosteroids are the major bioactive constituents in *P. angulata*. Only one P450 (PB.29059.4) was shown to be related to isoflavonoid biosynthesis, three P450s (PB.9773.5, PB2181.4, and PB24953.3) to alkaloid biosynthesis, three P450s (PB.12493.7, PB.30902.1, and PB.2636.75) to triterpenoid biosynthesis and four P450s (PB.8240.3, PB.23483.5, PB.31382.1, and PB.34288.1) to diterpenoid biosynthesis. The other 19 P450s, which were PB.19196.4, PB.20392.1, PB.34165.2, PB.21245.1, PB.29095.11, PB.26424.5, PB.29628.4, PB.33608.1, PB.17445.12, PB.21313.2, PB.29452.1, PB.4191.2, PB.23000.2, PB.28004.2, PB.21306.1, PB.30335.2, PB.24463.1, PB.30090.2, and PB.21064.10, all showed closer relationships to the ones involved in biosynthesis of steroids (Figure 3). Among the 19 P450 candidates, 11 candidates belong to the CYP94 family, four to the CYP734 class, one to the CYP710 family, one to the CYP90B family, one to the CYP85A family, and one to the CYP90A subclass. Obviously, the CYP94 and CYP734 family members expanded in *P. angulata*, suggesting their possible roles in the physalin biosynthesis pathway. Indeed, some members of the CYP94 and CYP734 families have already been reported to catalyze hydroxylation reactions during the steroid catabolism [17,18,19,20].

In order to validate the P450 candidates identified by this study, four P450 candidates (PB.34165.2, PB.26424.5, PB.11591.2 and PB.29095.11), which showed a comparable FPKM value to the *24ISO* gene presented in the transcriptome, were selected for the VIGS experiment. When the *24ISO* expression was down-regulated, the contents of physalin H and dihydrophysalin A almost halved in comparison with those of the control plants, suggesting that the VIGS experiments of this study were correctly performed. Interestingly, down-regulation of the PB.11591.2 candidate decreased the contents of both physalin H and dihydrophysalin A by more than 80%, allowing us to propose it as the best candidate associated with physalin biosynthesis. The closest homolog to PB.11591.2 is a hypothetical protein from *Datura stramonium*, a species that also produces abundant withanolide-related steroids [21], indicating that the PB.11591.2 is a novel P450 related to withanolide metabolism. The PB.26424.5 candidate belongs to the CYP734 A1 subfamily. Cotton CYP734A1 regulates fiber development through inactivating the levels of endogenous brassinosteroids via C-26-hydroxylation [22], suggesting that CYP734 A1 members are able to catalyze C-26-hydroxylation in steroid metabolism. A lactone moiety is formed between C-22 and C-26 in physalins (see Figure 1A), indicative of involvement of C-26-hydroxylation in their biosynthesis. Thus, the PB.26424.5 would be the likely candidate catalyzing the C-26-hydroxylation in physalin biosynthesis. The PB.34165.2 is a member of CYP90A1 family, which usually catalyzes C-22-hydroxylation in a brassinosteroid metabolism [23]. Therefore, we propose that the PB.34165.2 may be the P450 enzyme responsible for the C-22 hydroxylation in physalin biosynthesis. Indeed, the reduction in the *PB.34165.2* transcripts significantly decreased the contents of physalin H and dihydrophysalin A (Figure 4). In short, through extensive transcriptomic and phylogenetic analysis, this study provides a number of P450 candidates that are associated with physalin biosynthesis, constituting a valuable genetic resource for further elucidating the physalin biosynthetic pathway. It will be of particular interest to further investigate the biochemical functions of the P450 candidates identified by this study.

## 4. Materials and Methods

### 4.1. Plant Materials and Chemicals

The seeds of *Physalis angulata* were harvested from wildly grown plants in a field at the Langxi County, Anhui, China. The *P. angulata* plant was identified by Prof. Xiaodong Li at the Wuhan Botanical Garden, Chinese Academy of Sciences. *P. angulata* plants were grown in a growth chamber at 22 °C with a cycle of 16 h of light/8 h of darkness. The chemical standards of physalin A, physalin G, physalin D, physalin L, and isophysalin A were purchased from the ChemFaces Co. Ltd. (Wuhan, Hubei, China).

### 4.2. Phylogenetic Analysis of the P. angulata P450 Candidates

Gene function of the *P. angulata* transcripts was annotated by using a BLASTx (E-value < 1 × 10^−5^) search against NR, KOG/COG, Swiss-Prot, KO (KEGG Ortholog), Pfam, and GO databases. The annotation data was searched to retrieve the *P. angulata* P450s using cytochrome P450 as the key word. The amino acid sequences of P450s were aligned using ClustalW program (http://www.ebi.ac.uk/clustalW/). A phylogenetic tree was constructed through the neighbor-joining method using MEGA software (MEGA 6.0) [24].

### 4.3. Transient Suppression of the P. angulata P450 Candidates

To confirm the VIGS system previously developed for *P. angulata* [10], the *PaPDS* (phytoene desaturase) gene was cloned from *P. angulata* leaf, and used as a reporter gene (see the *PaPDS* cDNA sequence in Figure S1). The VIGS constructs of the genes of interest (GOIs) were prepared using the pTRV2 vector [10]. The primers used for preparation of the VIGS constructs are listed in Table S3. The VIGS infection of *P. angulata* leaves was performed as previously described [10] with small modifications. Briefly, the Agrobacterium culture suspension of the pTRV1 and either pTRV2-GOIs, or the empty vector pTRV2 as a control, was mixed together at a 1:1 ratio, and the culture mixture was then infiltrated into the leaves of four to six-leaf-stage seedlings by a 1 mL syringe. The infected plants were kept in darkness for 48 h, and then grown in a growth chamber with a cycle of 16 h of light/8 h of darkness. After around two weeks, the phenotype of newly grown leaves was monitored.

### 4.4. Real-Time PCR Analysis

Real-time RT-PCR was performed on a Bio-Rad CFX96^TM^ Real-Time PCR instrument (Bio-Rad Inc., Hercules, CA, USA) using TransStart Green qPCR SuperMix (Transgen), and data were calculated by the 2^−ΔΔCt^ method [25]. Primers used for real-time PCR are listed in Table S3, and melt curves showing the amplifying specificity of each set of the primers are shown in Figure S4. The gene codifying for the *P. angulata* glyceraldehyde-3-phosphate dehydrogenase (*PaG3PDH*) was used as a reference gene, and a similar amplifying efficiency observed for *PaG3PDH* and the targeted genes (Figure S5) confirmed its validity as an internal control. The PCR program consisted of an initial step of 94 °C for 30 s; 40 cycles of 94 °C for 5 s and 60 °C for 30 s; and then a dissociation stage of 95 °C for 10 s, 65 °C for 5 s and 95 °C for 5 s.

### 4.5. Phytochemical Analysis

The dried plant sample (20 mg) was extracted with 1 mL methanol under sonication (180 W, 40 kHz, 30 °C, 20 min). To normalize the variation in extraction efficiency between samples, fexofenadine at a final concentration of 200 ng/mL was included as an internal standard. The clear methanol extracts were obtained by centrifugation, and were directly used for the ultra-performance liquid chromatography mass spectrum (UPLC-MS/MS) analysis.

One microliter of the extracts was injected for the UPLC-MS/MS analysis using the analysis condition previously reported by Huang et al. [14]. LC-MS analysis was performed using a Q-Exactive Focus mass spectrometer, coupled with Vanquish UPLC system (Thermo Fisher Scientific Inc., Waltham, MA, USA). The Hypersil GOLD column (100 mm × 2.1 mm, 3.0 μm) (Thermo Fisher Scientific Inc., Waltham, MA, USA) was used to separate the sample. The column temperature was 40 °C, and the flow rate was 0.30 mL/min. The mobile phases contained 0.1% formic acid (solvent A) and acetonitrile (solvent B), and the solvent gradient was set as follows: 10 to 20% B (0–10 min), 20 to 30% B (10 to 40 min), and 30 to 50% B (40–48 min). The MS detection was performed in a negative electrospray ionization mode. The parameters of the mass spectrometers were as follows: spray voltage, 3.2 kV; source capillary temperature, 320 °C; sheath gas flow rate (nitrogen), 25 mL/min; Aux gas flow rate (nitrogen), 8 mL/min; Aux gas heater temperature, 30 °C, Scan range 120.0–1000.0 *m*/*z*. The mass data were processed with Xcalibur 4.4 software (Thermo Fisher Scientific Inc., Waltham, MA, USA) [17].

### 4.6. Statistical Analysis

Phytochemical data was shown as mean ± SD (Standard Deviation) of three biological replicates. Data analysis was performed by one-way ANOVA [26]. Difference was considered statistically significant when * *p* < 0.05 and highly significant when ** *p* < 0.01.

## 5. Conclusions

The pathway for the conversion of 24-methyldesmosterol to physalins has not yet been dissected. It is assumed that from 24-methyldesmosterol physalin is biosynthesized through multiple-step oxidations on different carbons catalyzed by P450s. A total of 306 putative P450-encoding sequences were retrieved from our recently constructed *P. angulata* transcriptome. Detailed phylogenetic analysis revealed 21 P450 candidates that might take part in physalin biosynthesis. Of those, we selected four P450 candidates for the test by suppressing their expression in *P. angulata* leaves, via the VIGS technique. Reduction in their transcripts significantly decreased the contents of physalin H and dihydrophysalin A, suggesting their roles in physalin biosynthesis. Thus, the 21 P450 candidates identified by this study will be valuable for further investigation to understand the downstream unknown pathways for physalin-class compounds.

## Data Availability

The raw data for the RNA-sequencing reads of *P. angulata* generated by us was deposited in the NCBI-SRA database under the BioProject ID of PRJNA986224. The original contributions presented in the study are included in the article/Supplementary Materials, further inquiries can be directed to the corresponding author/s.

## Supplementary Materials

The following supporting information can be downloaded at: https://www.mdpi.com/article/10.3390/ijms241814077/s1. References [27,28,29,30,31,32,33,34,35,36,37,38,39,40,41,42,43,44,45,46,47,48,49,50,51,52,53,54] are cited in the supplementary materials.
