# Supplementary material for: Identification of P450 Candidates Associated with the Biosynthesis of Physalin-Class Compounds in Physalis angulata
Source: Int J Mol Sci. 2023 Sep 14;24(18):14077. doi: 10.3390/ijms241814077 (PMC10531436; doi:10.3390/ijms241814077)
Supplement: Supplementary file 1 [file ijms-24-14077-s001.zip › ijms-2607106-supplementary.docx]

Supplementary Material

**Identification of P450 candidates associated with the biosynthesis of physalin-class compounds in *Physalis angulata***

Congkun Hua, Zhengqin Xu, Nan Tang, Yehan Xu, Yansheng Zhang, Changfu Li^*^

Shanghai Key Laboratory of Bio-Energy Crops, Research Center for Natural Products, Plant Science Center, School of Life Sciences, Shanghai University, Shanghai, 200444, China

*Correspondence: changfuli@shu.edu.cn (C.L.)

atgccccaaattggacttgtttctgctgttaacttgagaatccaaggtaattcagcttatctttggaggtcaaggtcttgtttgggaactgaaagtcaagatggttgcttgcaaaggaattctttatgttttggtggtagcgaatccatgggtcataagttaaaaagccctactccccttgccatgaacagaagattggctaaggacttgcggcctttaaaggtagtttgcattgattatccaaggccagagctagacaatacagttaactatttggaggctgcattcttatcatcatcattccgaacttctcctcgcccaactaaaccattggagattgttattgctggtgcaggtctgggtggtttgtctacagcaaaatatttggcggatgctggtcacaaaccgatactgctggaggcaagggatgttctaggtggaaaggttgccgcatggaaagatgatgatggagattggtacgagactggtttgcacatattctttggggcttacccaaatatacagaacctgtttggagaattagggattaacgatcgattgcagtggaaggagcattcaatgatatttgcaatgcccagcaagccgggagaattcagccgttttgatttccccgaagctttacctgctcctttaaatggaattttggccatcctgaaaaacaatgaaatgcttacatggccagagaaagtcaaatttgcaattggactgttgccagcaatgcttggagggcaatcttatgttgaagctcaagacggaataagtgttaaggactggatgagaaagcaaggcgtgccggatagggtgacagatgaggtgttcattgccatgtcaaaggcacttaactttataaaccccaatgagctttcaatgcagtgcatcttaatcgcgttgaacagatttcttcaggagaaacatggttcaaaaatggcctttttagatggtaatcctcctgagaggctttgcatgccgattgttgaacatatcgagtcaaaaggtggccaagtaagactaaactcacgaataaaaaagattgagctgaatgccgatggaagtgtcaaatgttttatcctgaacgatggtagtacaattaagggagatgcttttgtgtttgccactccagtggatattctcaagcttcttttgcctgaagactggaaagagattccatatttccaaaagctggagaagttagtcggcgtacctgtgataaatgtacatatatggtttgacagaaaactgaagaacacatatgatcatttgcttttcagcagaagttcactgctcagtgtgtatgctgacatgtctgtcacatgtaaggagtattacaaccccaatcagtctatgttggaattggtttttgcacctgcagaagagtggatttctcgcagcgactcagaaattattgatgctacaatgaaggaactagcaacgctttttcctgatgaaatttcggcagatcagagcaaagcaaaaatcttaaagtatcatgttgtcaaaactccaaggtctgtttataaaactgtgccaggttgtgaaccctgtcggcccttgcaaagatcgcctatagaggggttttatttagctggtgactatacaaaacagaaatacttagcttcaatggaaggtgctgttttatcgggaaagctttgtgcacaagctattgtacaggattatgagttacttgttggccggagccagaaggtggaggtggaagcaagcttagtttaa

**Figure S1.** The cDNA sequences of *PaPDS* retrieved from the *P. angulata* transcriptome*.*


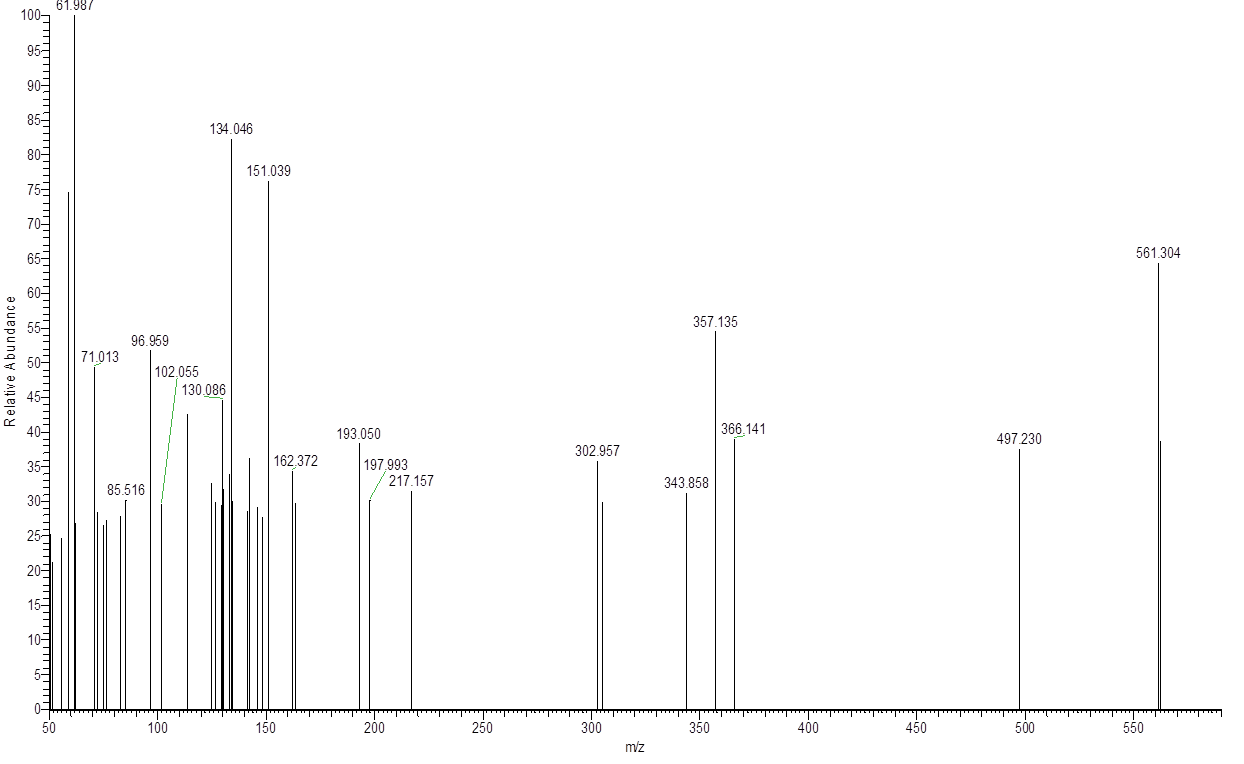


Diagnostic ions: 561，497，193，135，129，71

**Figure S2**. Mass fragmentation pattern of physalin H produced by the *P.angulata* leaf


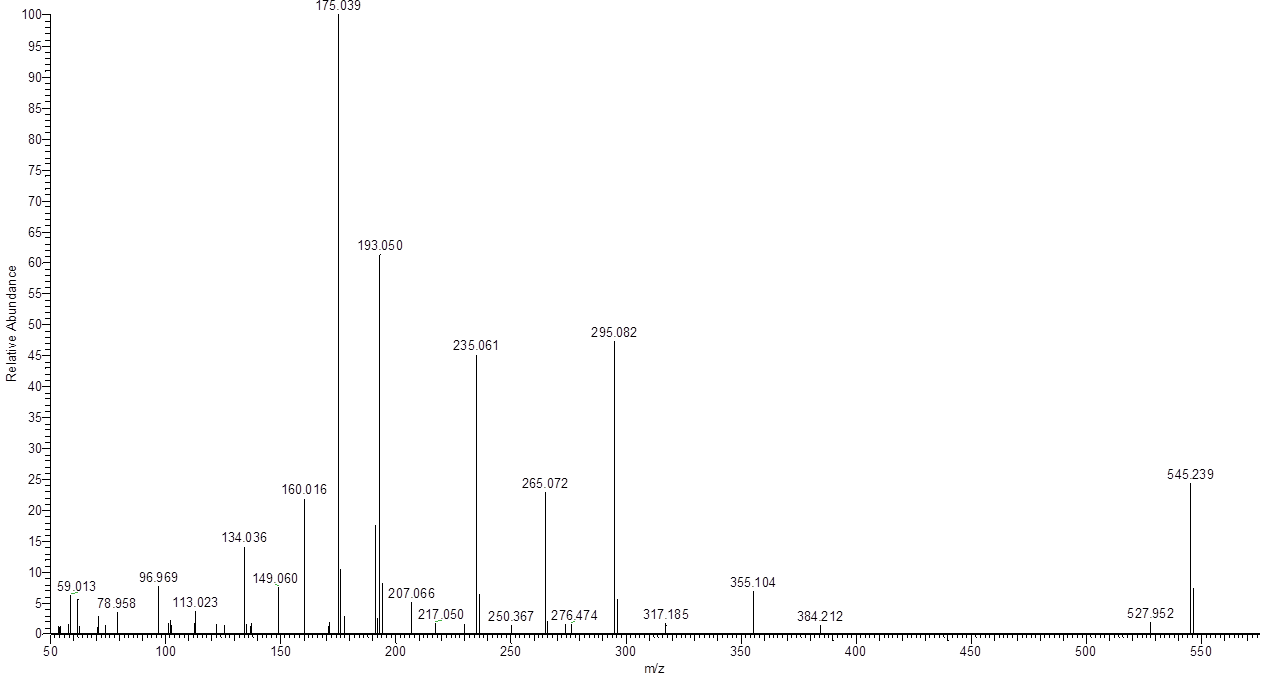


Diagnostic ions: 545，527，277，149，121

**Figure S3**. Mass fragmentation pattern of dihydrophysalin A produced by the *P.angulata* leaf


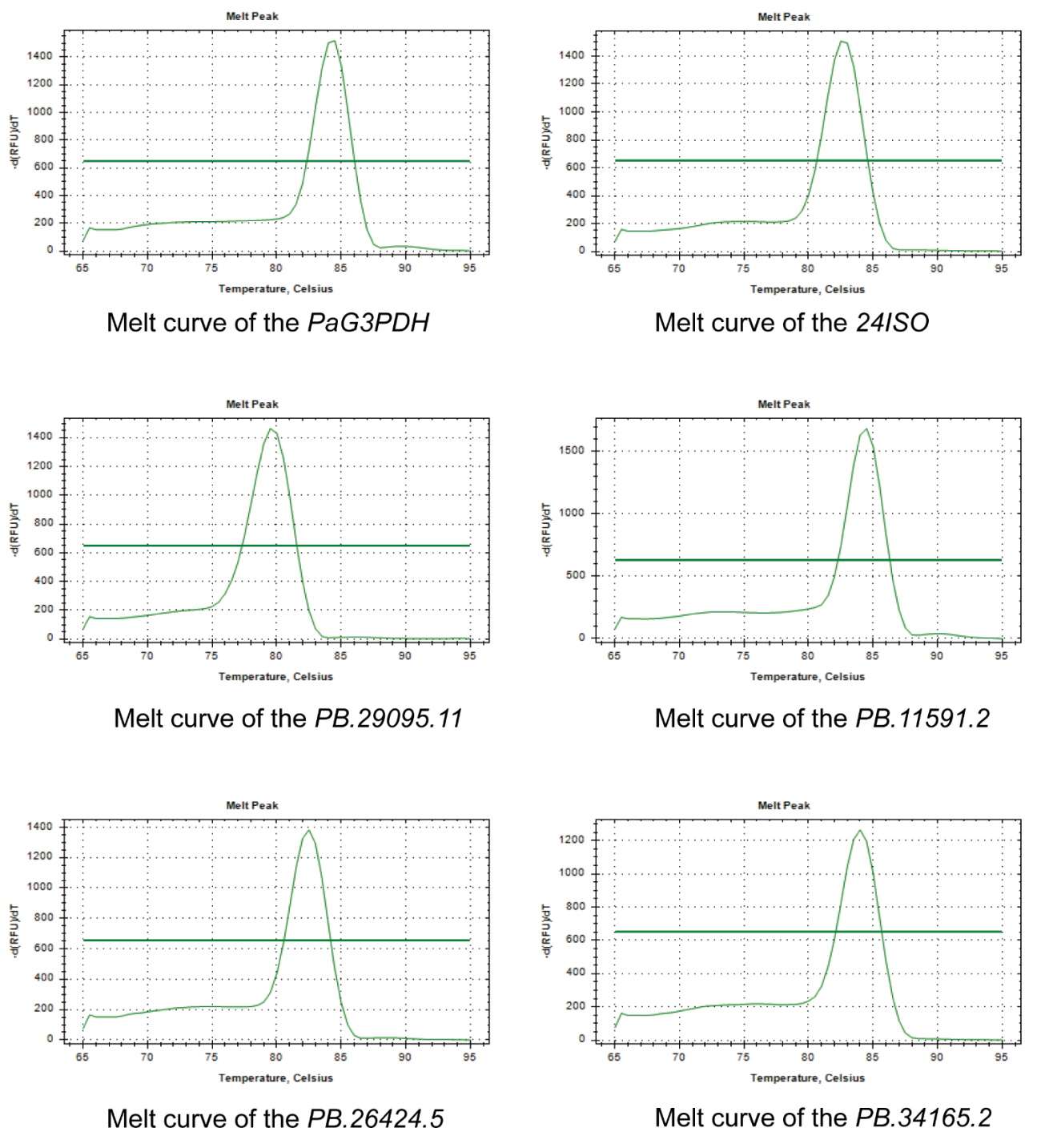


**Figure S4**. The melt curves showing the amplifying specificity for the primers used to amplify *PaG3PDH*, *24ISO*, and the targeted P450 candidates.


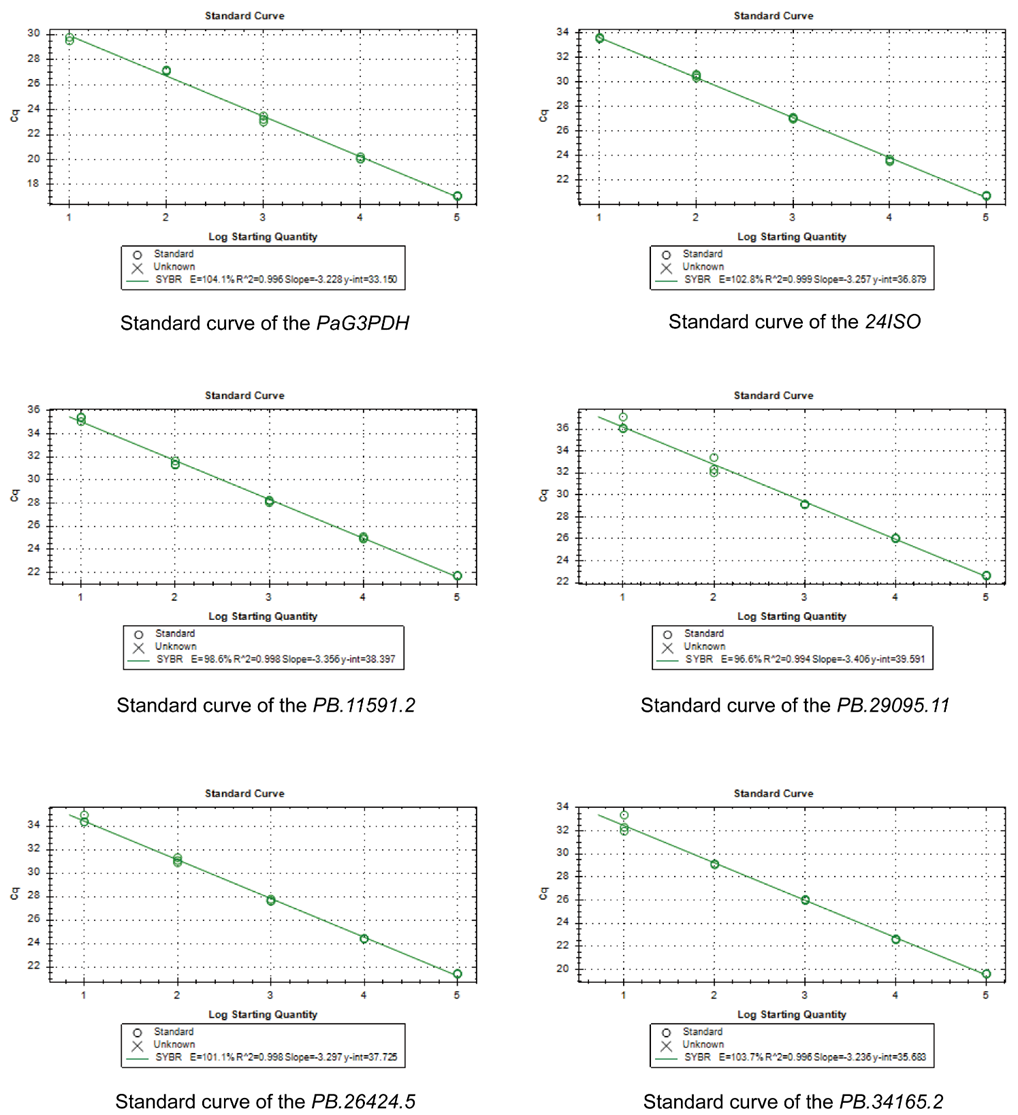


**Figure S5**. The standard curves showing a similar amplifying efficiency for the internal reference gene *PaG3PDH* and the targeted P450 transcripts.

**Supplemental Tables**

**Table S1**. Accession number of the previously characterized P450s selected for the phylogenetic analysis of this study

| **Name** | **GenBank accession number** | **Biochemical pathway** | **References** |
| --- | --- | --- | --- |
| CYP734A | BAF56240 | Steroid biosynthesis | [1] |
| CaCYP72A565 | A0A4Y5UJ61 | Alkaloid biosynthesis | [2] |
| CaCYP72A610 | A0A4Y5UJ78 | Alkaloid biosynthesis | [2] |
| CrCYP72A224 | U5NE19 | Alkaloid biosynthesis | [3] |
| CrCYP72A1 | Q05047 | Alkaloid biosynthesis | [4] |
| CYP85A2 | NP_566852 | Steroid biosynthesis | [5] |
| CYP85A1 | XP_019067501 | Steroid biosynthesis | [6] |
| CYP85A3 | NP_001234520 | Steroid biosynthesis | [7] |
| AtCYP708A2 | Q8L7D5 | Triterpenoid biosynthesis | [8] |
| CYP724B1 | NP_001389305 | Steroid biosynthesis | [9] |
| CYP90B71 | MN829441 | Steroid biosynthesis | [10] |
| PtCYP720B1 | Q50EK6 | Diterpenoid biosynthesis | [11] |
| SsCYP720B4 | E5FA70 | Diterpenoid biosynthesis | [12] |
| PtCYP720B2 | Q50EK5 | Diterpenoid biosynthesis | [13] |
| PgCYP720B12 | A0A0G7ZP11 | Diterpenoid biosynthesis | [14] |
| CYP90A1 | NP_001291222 | Steroid biosynthesis | [15] |
| CYP90C1 | KAG6594802 | Steroid biosynthesis | [16] |
| CYP90D2 | NP_001396000 | Steroid biosynthesis | [17] |
| AtCYP88A3 | O23051 | Diterpenoid biosynthesis | [18] |
| AtCYP88A4 | Q9C5Y2 | Diterpenoid biosynthesis | [18] |
| TwCYP88A43 | A0A1X9H7V5 | Diterpenoid biosynthesis |  |
| GuCYP88D6 | B5BSX1 | Triterpenoid biosynthesis | [19] |
| CYP710A | NP_180997 | Steroid biosynthesis | [20] |
| AtCYP701A3 | Q93ZB2 | Diterpenoid biosynthesis | [21] |
| OsCYP701A6 | Q5Z5R4 | Diterpenoid biosynthesis | [22] |
| OsCYP701A8 | Q0DBF4 | Diterpenoid biosynthesis | [23] |
| EcCYP719A2 | Q50LH3 | Alkaloid biosynthesis | [24] |
| EcCYP719A3 | Q50LH4 | Alkaloid biosynthesis | [25] |
| PsCYP719B1 | B1NF18 | Alkaloid biosynthesis | [26] |
| DcSLS1 | A0A2I0W3Y | Alkaloid biosynthesis |  |
| DcSLS2 | A0A2I0WQE | Alkaloid biosynthesis |  |
| DcSLS3 | A0A2I0XA89 | Alkaloid biosynthesis |  |
| DcSLS4 | A0A2I0WQG7 | Alkaloid biosynthesis |  |
| CYP94N8 | MN829443 | Steroid biosynthesis | [27] |
| CYP94D143 | MN829444 | Steroid biosynthesis | [27] |
| CYP81E7 | Q6WNR0 | Isoflavonoid biosynthesis | [28] |
| CYP81E | Q9MBE4 | Isoflavonoid biosynthesis | [29] |
| GeCYP81E1 | P93147 | Isoflavonoid biosynthesis | [30] |
| CYP81E3v1 | Q9ZRW6 | Isoflavonoid biosynthesis | [31] |
| CYP81E3v2 | Q9XFX0 | Isoflavonoid biosynthesis | [31] |

**Table S2**. Predicted amino acid sequences of the 21 P450 candidates identified by this study

| No. | Gene ID | Putative amino acid sequences |
| --- | --- | --- |
| 1 | *PB.11591.2* | mipsdlvfdftwklvavvlmvllvrgfwrtyvskfsfmygnredvetdveagpvprtpllslryslhhapmlsansdtklamkgishgacdylvklvrieelrniwqhiirrkkvepkmeynlvflstafavgiltlisvlkrangwfysmkfssekcrlppgdmgwpvvgnmlffvkclsaydlksfvsyfvtrfgqggmyrtfmfgkpsvivttpelcrkilmddenfdlgfpsyilellrkepiggtsyqedrlsrrlmtpikshalvsyffdflsetvqttfekwattgeslqllfemkkptfkvlmqvliggdqvenklldtlfkennfrfaglrslpldypgstynramkgrgeivkiyeriinerkvmiaktrgeprtnlldimldsqydgegkvlndenimkvllwytfsgyesiakvatqtimllekhpecfkkakeeqeeivkrrsspdagltfgeigqmkyvnnvinetlrlgstetvlfrdartdvningytipkgwkvlallgnlymdpktyvkpkefnpsrwddfetkpnsfipfgvglrlcpgsnlvrlevsvflhyfllnyrleqlnkdskaeaciakfkkisa |
| 2 | *PB.2636.75* | mwniilcivglvvvgithwvyrwrnpkckgvlppgsmglpligetlqyfskspyegippfiaerkakygtlfktslvgqpiiistdpdvnyyvfqqedklfqcsytksavelsgkkglmgnggsahkylrnlvlsltgpdklktklvsdvdiitrehlhrwttqgdvevkdaseimlfifiaakilgmnekealtlrghykafvkgflsfpinlpgtafhsglqgrknaikmikdifekrrsskdkaneqdfidhllqeidkedtfitedtavdliffvifaahettsstmtllfkyftehpdvvkklkeeheniirnredknapiswseyksmnfthkvtnetvrlaniapgifrkvlkdveikgytipegwtmvvcspsvhldenmydnplafnpyrwkddelgaskkfmafgggnrmcvgadlskmqtsifihylmtsfkwkvaskgniirqpylnfndgicirvdeiqrdn |
| 3 | *PB.34165.2* | mdtinlflyvflsvftflllrgmaavhlrrrktqlppgtlglpfigetlqliaayktenpepfiddrvskygniftthvfgeptvfsadpetnrfilqnegrlfessypgsiqnllgrhslllmrgtlhkrmhsltmsfanssilkdhlladidrlvrlnmdswtarvflmeeakkitfnltvkqlmsfdpcewtenlmkeymlviegfftvplpffsstyrkaiqarrkvaealslvvrgrrkerdggerkndmlealfegdgvegggfsdeeivdfmlallvagyettstimtlavkfltetshalshlkeeheeirlrkgeveslqwedyksmpftqcvvnetlrianiiggvfrramtdinikgyiipkgwkvfasfravhldhehfkdartfdpwrwqsnagltsspnlftpfgggprrcpgyelarvelsvflhhlvtrfswvpaepdklvffpttrmqkrypiiirhrslfdacske |
| 4 | *PB.21064.10* | vdgdiwkyqrqvashefntrslrkfvetvvdtelserlvpilanaaanktvvdlqdilqrfafdnickiafgydpgyllpslpeaefavafedsvrlssqrfmlpfpliwkikralnigserklriavqqvrefakkivrekqrelkekssldsadmlsrflsqghsdedfvtdivisfilagrdttsaaltwffwliwknprveseilkeigekadgslvlydnvkemmythaslcesmrvyppvpmdskeavkddvlpdgtfvkkgtrvtyhpyamgrseeiwgkdcgefkperwlmnkddegrgnnwvfvskdayaypvfqagprvclgkemaflqmkrvvagvmkrfkvvpvntenpmfisyltakmkggfpvtiqeri |
| 5 | *PB.30335.2* | mdivyaiftcivvllficvtpilvlivriysgksirnpnyapvvgtvfhqllyfhriydyqaelakkaptfrllgpeqsetyttdsrnvehilktnfgiyskgkrnqevimdlfgegifavdgekwkqqrklasfefsarvlrdfsctvfrqratklvskvfefalanqdfdmqellmrcsldsifkvgfgvdlncldgssgdnnkfikafddsnalsywryvdpfwklkryfnigsefllkknikfihifvdgliktrrkqlemkqvsmdkedilsrflveskkdpekmtdeylrdiilnfmlagkdstantlswffyvlcknpliqakvveeirevignnmnnngnaedfvaaiteevlekmhylhatltetlrlypavpvdgrcadaddilpdgfhirkgdgvyymsyamgrmtyiwgndaedfrperwlkdgifqpespfkfiafhagpriclgkdfayrqmkilsmallhffrfklsddtkvvtyrtmftlhineglqvcafprrglvea |
| 6 | PB.21245.1 | masiwvlispwtpylfsfiallllleqilylkkkrflpgpslvipffgsvfslvtnptkfwdlqsslakstkhgfsanyiigkfilyiqstdlshkvfanvrpdafhlighpfgkklfgehnliymfgqehkdlrrriapnftpkalatytdiqqrimikyfkswldqaskspntpiplrflcrdmnldtsqtvfvgpylneesrkqfnvdynyfnvglmklpfdfpgfafrdarlavgrlvetlsvcaeqslnkmqsneeptclidfwmqenlreieeakinglqkpfeytnkelggylfdflfaaqdastsallwaivlldshrevlervraevarfwspeseqpltgemlremkyleavareivrirapatmvphiageefrltedyvipkgtivfpsvfdssfqgfpepekfdpdrfmeerqeervykknflafgagphacvgqkyainhlmlfiaiftalidfnrhktdgcddisyiptiapkddckvflsqrctrfpsls |
| 7 | PB.21313.2 | mdivialflftaitcyllwftfisrslkgprvwpllgslpgliensenmhewivdnlsacggtyqtcicaipflarkqglvtvtcdpknlehilktrfenypkgptwqavfhellgqgifnsdgdtwlfqrktaalefttrtlrqamarwvnraiklrfcpilktaqlegkpvdlqdlllrltfdnicglafgkdpqtlapglpdntfasafdrateaslqrfilpeviwklkkwlglgmevslnrslvqldkymsdiintrklelmsqqkdgnphddllsrfmkkkesytdkflqhvalnfilagrdtssvalswffwlviqnpaveqkilqeictvltetrgsdtsswlaeplafeevdqltylkaalsetlrlypsvpedskhvmvddvlpdgtfvpagsaitysiysagrmkatwgedclefkperwlsedgkkfvmheqykfvafnagpriclgkdlaylqmksvaaavllrhrlsvapgheveqkmsltlfmkdglkvdlrprdltpfvnsvkkerevelvlqk |
| 8 | PB.30090.2 | mavstlfflilavlvlvlsalfflsrikpyceceickgylnstwslefknlcdwyshlltksptgtihvhvlgnvitanpknveymlktkfenfpkgkqfstivgdllgrgifavdgemwkfqrkmaslelgsvsirsyafdivgdeirnrlipllesnvrngavldlqdvlrrfsfdsickfsfgmdpgclklslpvsdlqvafdlasklsaeramtvsplvwkikrvlnigsekklkeaiklvdilaaevinhkrkndfssqddllsrfmrsidddkllrdivvsfllagrdtvasalttffwllgqhpqvidgiraessrvmggkrkattpfatfeeiremhyltaaihesirlfppvqfdskfcqdddtlpdgtfvakgtrvtyhpyamgrmesiwgqdclefkperwldddglfkpqcpfkypvfqgrvrvclgkdlaivemksvalalirqfdfevvakeqtpkfmpgltatvrgglpvmvrerrrqq |
| 9 | PB.21306.1 | mrcsldsifkvgfgvdlncldgssgddnefikafddanaltywryvdplwklkrffnigsefflkknikfirefvdelirtrrkqleikqdsmdkedilsrflveskkdpekmtdeylrdiilnfmlagkdstantlswffsvlcknpliqvkiveeirevignnmkdnlsvndfvaaiteqvlekmhylhatltetlrlypavpvdgrcadaddvlpdgfhirkgdgvyymsyamgrmtyiwgddaedfrperwlkdgifqpespfkfiafhagpriclgkdfayrqmkilsmallhffrfklsddtkvvtyrtmftlhineglqvravprrglvea |
| 10 | PB.33608.1 | mylilvailllfpvtlvvkliysviyipwkfekhfrkqgirgpgyrliygnseeikrqiseaeskpvplnhnvlhriaphyynwsamygktflwwfgskprlaisdpdmikglfmnkavdkiefnpqskmlfgdglvglkgeewalhrrianqafnmeivkawvpdivasvikvlkkwekeneekeefevevfkelndlsaevisrtafgssfeegkrifvlqeqqisltlqalrsiylpgfsylptknnmmrrrleketlesvrklieisskgrensknllsllisaseeehgfgmdavidecktfyfagkettsnlltwallllalhqewqdkareevfrvckgnnlptaenlndfkivtmilnetlrlyppvvaltretskdiklgdleipantqfyvslaavhhdteiwgedalefnpqrfseprkhlasyfpfalgprvcvgqnlamveakiilamivqnfsfalspsyvhaptmrltlqpqygapilfrki |
| 11 | PB.19196.4 | vksipfrlslsvrwfwifldfelhpmafflvflasffglcifstallrwnqvkynnknlppgtmgwplfgetteflklgpsfmknqrarygsffkshilgcptivsmdpelnryilvneakglvpgypqsmldilgkcniaavngsahkymrgallslisptmirdqllpkidefmrshltnwdnqvidiqektnkmaflsslkqiagiesssfaqefmpeffklvlgtlslpinlpntnyhrgfqarkiivrllgtlieerraskqihhdmlgymmneeanrfkltddemidliitilysgyetvsttsmmavkylhdhpkvleelrkehmairekkkpddpidyndykamrftraviletsrlaivvngvlrkttrdmeingyiirggkiscksclnftyhy |
| 12 | PB.20392.1 | msdlefflflvppilavviilnlfkrkqkypnlppgdmgwpflgetigylrpysattigdfmqdhisrygkiyksnlfgeptivsadaglnryilqnegrlfecsyprsiggilgkwsmlvqvgqmhrnmrmislnflsnarlrnqllsevekhtllvlsswkhdsvvlaqdeakkftfnfmaehimslqpgnpeteqlkkeyitfmkgvvsaplnfpgtayrkalqsrstilgfierkmeerlkeingnekdllgwvlknsnlskeqildlllsllfaghetssvaialsiyflescpaavqqlkeehleisrakkqsgetelnwddykkmeftqcvinetlrlgnvvrflhrkavkdvrykgydipcgwkvlpvisavhldpllfdrphnfdpwrwqnkqgspsvnggstgmsstekscnnfmpfgggprlcagselaklemaifihylllnfhwqlaapdqafaypyvdfpnalpitiqttqinhhttpispyss |
| 13 | PB.30902.1 | meynlvflstafavgiltlisvlkrangwyysmkfssekcrlppgdigwpvvgnmlffvkclsaydlksfvsyfvtrfgqggmyrtfmfgkpsvivttpelcrkilmddenfdlgfpsyilellrkepiggtsyqedrlsrrlmtpikshalvsyffdflsetvqttfekwattgeslqllfemkkptfkvlmkvliggdqvenklldtlfkennfrfaglrslpldypgstynramkgrgeivkiyeriiierkvmiaktrgeprtnlldimldsqydgegkvlndeiekahpdifnillqlfedghltdsqgrrvsfknalivmtsnvgstaivkggqnsigffladdesaasyagmkaivmeelktyfrpellnridevvvfrplekpqmleilnlmlqevrarlvslgisldvseavmelicqqgfdrhygarplrravtqmvedllsesvlsgdfkpgdvamihldesgnpvvtnksshsihlsdsngnpvvtnr |
| 14 | PB.28004.2 | mftvetdgkqaltrthntlqvfmdihqssiisiaatiisllfsgfivhhltkrlvskngkkryhpvggtifnqllnfhrlhhymtdlaakyrtyrlitpfrneiytsdpanveyilktnfdnygkgcyhysnlkdllgdgiftvdgdkwreqrklsshefstrvlrdfssvafrknvaklahilseaasfkkavdiqdlfmkatldsifrvafgveldsmcgsneegknfsnafdnasemtlwryvdifwkikralniaseaklrdnirtvdefvyklirrkteqmskpeadlslqwkkedilsrflqitgtdqkflrdiilnfiiaskdttaatlswfiyvlckyphvqekvaqeikeatiekedetditdfaanvsedalekmqylhaaltetlrlypavpvdgkicfsddtlpdgfsvnkgdmvsyqpyamgrmkfiwgddaekykperwldgdglfrqespfkftafqagpriclgkefayrqmkifsavllryfvfklsddkktvnyrtminlhidgglpihvfhrsgh |
| 15 | PB.23000.2 | mdarsipttltmttlavilaavcvfklifsrsankgnyhpfagtgidhlknfdrlydymkemasnyktyrilylfrsevytsdpanveyilktnfpnygkgwhhydklndllgdgiftvdgekwrnqrkissyefstknlrdfssavfrtsavklaqkvsesvtsnqsmeiqdlfkrsaletvfkillgvdldttteegslfsssfdeasavtlyryvdgfwkvkrflniglesnmkkcikvvdefvykiirnkteqmiksqddsrvmtksdilsrflemnktnpkylkdiilsfiiagkdttastlswffymmckhpllqeriaeevreatrtnqncsvdelansitdealdkmqylhasltetlrlypavpvdgkvclsddtlpdgfrvrkgeavayqpwamgrmkflwgddaedfrperwfdengcfqqespfkftafqagpriclgkefayrqmkifsavllgtyrfklsdeerrvkyrtmltlhidgglhlhasyrlddvinmaaqlpy |
| 16 | PB.17445.12 | mevstgmmivaivvgylvwfksitksmkgpkgpktwpivgslpgllengnrmhewiaenlrvctgtyqtcicaipflarkqglvtvtcdpknlehilkvrfdnypkgptwqavfhdllgegifnsdgdtwlfqrktaalefttrtlrqamgrwvnraikdrfcpiletaqvqgkpvelqdlllrltfdnicgltfgkdpetlspglpnnvfatsfdrateatlhrfiipefvwklkkmlglgmevslshslrqlddymtdvintrklelvnhqngvgpqhddllsrfmkkkesysdkflqhvalnfilagrdtssvalswffwlvslnprveekilvelctvlvetrgndtskwldeplvfeevdqltylkaalsetlrlypsvpedskhvisddylpdgtfvpggsnitysiystgrmkfiwgedclefkperwmsqdgnkyqvqdafrfvafnagpriclgkdlaylqmksiaaavllrhrlvvapghkveqkmsltlfmkyglvmnvtprdltpvlakigihqpaii |
| 17 | PB.29452.1 | mdpillyfgivaattayflwfwllvqrlngpkvwplvgslpynflnrrrfhdwisqnlrstggaatyqtcticipflawkqgfytvtchpkniehilrtrfdnypkgpnwqnafddllgqgifnsdgdtwliqrktaalefttrtlrqamnrwvnrtirtrlwvildkaakektpvelqdlllrltfdnicgltfgkdpetlspkmpenpfaiafdsateatmqrllypyflwrlkkflgigaekrlqkslkvvenyisealdsrkespsddllsrfmkkkdingnsfpsdvlkrialnfvlagrdtssvalswffwnvmnnchvenkiveeistvlkgsrgenyekwieeplnfdeadklvylkaalaetlrlypsvpedfkyvvsddvlpdgtwvpagstvtysiysvgrmktvwgedcmefkperwistggdrfeppkdgykfvafnggprtclgkdlaylqmksvaaaillryrllpvpghkveqkmsltlfmknglkvylhprelapkia |
| 18 | PB.4191.2 | mdvlgyslipliiilcftywyfkntstkssqptnwplagmlpglvhnahrihayftdilletssnfefrgpvfaqmdmlftsdpanihhilsrnfsnypkgpefrkifdvlgngifnvdselwevhrkttmplfnhakfqvslqrnvwdtidkglvpvldtfakqdnlvvdlqdifqrftfdaiskllldhdpkslsvnlpivpcekafndmvdalvyrhvlpesfwklqkwlrigreknlseaweafdrfiypairkkqeklsnrinkdedqdfddlftdfvkaynqwstnygdgdsnlgsvqdflrdtflnlmfagkdttstaltwffwllaknplvekkireeiqqqlrlsddeklkffnveesrklvylhgalcealrlfppvglehkapleldvlpsghrvspntkilisfytmgrmeslwgkiv |
| 19 | PB.24463.1 | mdhlesfhvysililmvlslfslifyivriklwcnceichayitrswssqfinlcdwythllkksptrtihihvlgniitsnpqnveymlktrfenypkgktfstilgdflgrgifnvdgdswrfqkrmsslelgkvsirsyafevvkneidkrlipllddykqggvlldlqdvfrrfsfdsicrfsfgldpkclesslpisqfalsfdlasklsakramttspivwkikrflnigsekelreaikvinilakevirqkrklgfsnhrdllsrfmgsisdetylrdivisfllagrdtiasaltsffyvianhpqvaksirdeadrvlgpnkdltsyeqmselhylqasiyesmrlyppiqfdskfcleddflpdgtfvkkgtrvtyhpyamgrmdelwgcdslefkperwlrngiffqenpfkypvfqagprvcvgkemalvelksvalslirrfhvelshpfhhaprfspgltasfnggllvsvqkislsvvdvdse |
| 20 | PB.29628.4 | mffltlvfisllltlkvvhkiiwvplkvqhefkkqgikgpgyspilnntakirrvmiaeaesnstpfthhhelvvsrvmphyynwsmiygknflywfgpkprlaiadpnlikdillnttasfekvkhnplsnlllgdglvgldghkwalhrritnqafnmetvkgwipemvesttkllekweevgsardqfemdvhrefhtlsadiisrtafgssfeegkrifelqdqqvslvleairsvyipgfrflptkknrlrsrleketrdaikklikncsrtanhsksllsllmyplknqgdeketldieevvdecktfyfagkettanfltwaflllalhqewqskareevvracrngiptaeniadlkivsmilnetlrlyppavmlmrqaskkvqlgnldipantqfylamtavhhdkeiwgddahefnplrfaeprkhlasffpfglgpricvgqnlalieakvvlamiiqqysfcispsyihaptqamtlqpqygaqilfskic |
| 21 | PB.26424.5 | megfcfqwlffvviwyvlllfaqrafvhlwltpkriqkhfknqgitgpkyhflfgnlkeissfttctpswpspfishhdiiptvlpfyhhwkkiygsifviwfgptarvtisdpalirdifvqksdnfeknespalvkklegdgllslkgekwahhrkiitptfyienlklmipvmgksmtemldkwskmsnasgkveievsemfstlsedvitrivfgssyedgraifelqaqqmvyateayqkvfipgyrflptkknricwrldkevrkslmklieerrsklwssddgqvlseecpndllevmikasthyvddfeytnnmtvndiveecktiffagkhttsnlmtwttillamhpqwqelareevlsvcgardppskdhlsklktlgmiinesvrlyppavaairrakvdtqlgdfrlpkgtellipiiaihhdqtlwgedanefnparfgrgvaqaakhpmafmpfglgarrcigqnlavlqaklaiamilqrfsfdlspsyqhapsilmllcpqygapvifqkl |

**Table S3**. Primers used in this study

| No. | Sequence (5’-3’) | Gene ID | Description |
| --- | --- | --- | --- |
| 1  2 | GTGAGTAAGGTTACCGAATTCTGGAGAAGTTAGTCGGCGTACC  GGCCTCGAGACGCGTGAGCTCAAGCGTTGCTAGTTCCTTCATTG | *PaPDS* | For preparation of the VIGS construct |
| 3 | GGAATTCCCATCCTCAAGGACCATCTGTTG | *PB.34165.2* | For preparation of the VIGS construct |
| 4 | CGAGCTCGGTGAGGAATTTGACAGCTAATGTC |  |  |
| 5 | GGAATTCCGTAGTGATGCATATGGAAGGCTTC | *PB.26424.5* | For preparation of the VIGS construct |
| 6 | CGAGCTCGTGAGCAAACCATCACCTTCAAGC |  |  |
| 7 | GGAATTCCGTGTGCTAAAGAGAGCAAATGG | *PB.11591.2* | For preparation of the VIGS construct |
| 8 | CGAGCTCGTGAAGAGATTCTCCAGTTGTAGC |  |  |
| 9 | GGAATTCCCTCGAAATCCTATTACCGAGAAAC | *PB.29095.11* | For preparation of the VIGS construct |
| 10 | CGAGCTCGGTAGAATGAATGGGGGATAGAGC |  |  |
| 11  12 | GGAACTGAAAGTCAAGATGGTTGC  GATGATGATAAGAATGCAGCCTCC | *PaPDS* | For real-time PCR |
| 13 | GGTCCATCTTCGTCGGCGTAAA | *PB.34165.2* | For real-time PCR |
| 14 | TTCGGTCTTGTAGGCGGCAATT |  |  |
| 15 | CGTGAGGAAGTCTTGAGTGTCTGT | *PB.26424.5* | For real-time PCR |
| 16 | TCTGATTGCTGCCACCGCTG |  |  |
| 17 | TAGAGGAAGCCAAGGACCAACT | *PB.11591.2* | For real-time PCR |
| 18 | GTGTGGCAACCTTAGCAATAGATT |  |  |
| 19 | ATTGGAGCACTGGTTCTTGGTAGG | *PB.29095.11* | For real-time PCR |
| 20 | GTTCCCACTGCCAAATTCGTAAGG |  |  |
| 21 | GGTTGTCTCTA GCAGAGACCCTC | *PaG3PDH* | For real-time PCR |
| 21 | CGCGACCTCGTGAGAGTAGTC |  |  |

References

1. Thornton, L.E.; Peng, H.; Neff, M.M. Rice CYP734A cytochrome P450s inactivate brassinosteroids in Arabidopsis. *Planta* **2011**, *234*, 1151-1162, doi:10.1007/s00425-011-1464-2.

2. Yang, Y.; Li, W.; Pang, J.; Jiang, L.; Qu, X.; Pu, X.; Zhang, G.; Luo, Y. Correction to Bifunctional Cytochrome P450 Enzymes Involved in Camptothecin Biosynthesis. *ACS Chem Biol* **2021**, *16*, 1298, doi:10.1021/acschembio.0c00999.

3. Salim, V.; Yu, F.; Altarejos, J.; De Luca, V. Virus-induced gene silencing identifies Catharanthus roseus 7-deoxyloganic acid-7-hydroxylase, a step in iridoid and monoterpene indole alkaloid biosynthesis. *Plant J* **2013**, *76*, 754-765, doi:10.1111/tpj.12330.

4. Takemura, T.; Ikezawa, N.; Iwasa, K.; Sato, F. Molecular cloning and characterization of a cytochrome P450 in sanguinarine biosynthesis from Eschscholzia californica cells. *Phytochemistry* **2013**, *91*, 100-108, doi:10.1016/j.phytochem.2012.02.013.

5. Kim, T.W.; Hwang, J.Y.; Kim, Y.S.; Joo, S.H.; Chang, S.C.; Lee, J.S.; Takatsuto, S.; Kim, S.K. Arabidopsis CYP85A2, a cytochrome P450, mediates the Baeyer-Villiger oxidation of castasterone to brassinolide in brassinosteroid biosynthesis. *Plant Cell* **2005**, *17*, 2397-2412, doi:10.1105/tpc.105.033738.

6. Joo, S.H.; Kim, T.W.; Son, S.H.; Lee, W.S.; Yokota, T.; Kim, S.K. Biosynthesis of a cholesterol-derived brassinosteroid, 28-norcastasterone, in Arabidopsis thaliana. *J Exp Bot* **2012**, *63*, 1823-1833, doi:10.1093/jxb/err354.

7. Jin, Y.L.; Tang, R.J.; Wang, H.H.; Jiang, C.M.; Bao, Y.; Yang, Y.; Liang, M.X.; Sun, Z.C.; Kong, F.J.; Li, B.; et al. Overexpression of Populus trichocarpa CYP85A3 promotes growth and biomass production in transgenic trees. *Plant Biotechnol J* **2017**, *15*, 1309-1321, doi:10.1111/pbi.12717.

8. Tohge, T.; Fernie, A.R. Co-Regulation of Clustered and Neo-Functionalized Genes in Plant-Specialized Metabolism. *Plants (Basel)* **2020**, *9*, doi:10.3390/plants9050622.

9. Yan, B.; Zhang, Y.; Li, J.; Fang, J.; Liu, T.; Dong, L. Transcriptome profiling to identify cytochrome P450 genes involved in penoxsulam resistance in Echinochloa glabrescens. *Pestic Biochem Physiol* **2019**, *158*, 112-120, doi:10.1016/j.pestbp.2019.04.017.

10. Wang, W.; Hou, L.; Li, S.; Li, J. The Functional Characterization of DzCYP72A12-4 Related to Diosgenin Biosynthesis and Drought Adaptability in Dioscorea zingiberensis. *Int J Mol Sci* **2023**, *24*, doi:10.3390/ijms24098430.

11. Geisler, K.; Jensen, N.B.; Yuen, M.M.; Madilao, L.; Bohlmann, J. Modularity of Conifer Diterpene Resin Acid Biosynthesis: P450 Enzymes of Different CYP720B Clades Use Alternative Substrates and Converge on the Same Products. *Plant Physiol* **2016**, *171*, 152-164, doi:10.1104/pp.16.00180.

12. Hamberger, B.; Bohlmann, J. Cytochrome P450 mono-oxygenases in conifer genomes: discovery of members of the terpenoid oxygenase superfamily in spruce and pine. *Biochem Soc Trans* **2006**, *34*, 1209-1214, doi:10.1042/BST0341209.

13. Zhang, Y.N.; Ding, X.Y.; Luan, Q.F.; Jiang, J.M.; Diao, S. Identification and Tissue-Specific Expression Analysis of CYP720B Subfamily Genes in Slash Pine and Loblolly Pine. *Forests* **2022**, *13*, doi:ARTN 28310.3390/f13020283.

14. Geisler, K.; Jensen, N.B.; Yuen, M.M.S.; Madilao, L.; Bohlmann, J. Modularity of Conifer Diterpene Resin Acid Biosynthesis: P450 Enzymes of Different CYP720B Clades Use Alternative Substrates and Converge on the Same Products. *Plant Physiology* **2016**, *171*, 152-164, doi:10.1104/pp.16.00180.

15. Gan, Q.Q.; Luan, M.B.; Hu, M.L.; Liu, Z.S.; Zhang, Z.Q. Functional study of CYP90A1 and ALDH3F1 gene obtained by transcriptome sequencing analysis of Brassica napus seedlings treated with brassinolide. *Front Plant Sci* **2022**, *13*, doi:ARTN 104051110.3389/fpls.2022.1040511.

16. Ohnishi, T.; Szatmari, A.M.; Watanabe, B.; Fujita, S.; Bancos, S.; Koncz, C.; Lafos, M.; Shibata, K.; Yokota, T.; Sakata, K.; et al. C-23 hydroxylation by Arabidopsis CYP90C1 and CYP90D1 reveals a novel shortcut in brassinosteroid biosynthesis. *Plant Cell* **2006**, *18*, 3275-3288, doi:10.1105/tpc.106.045443.

17. Sakamoto, T.; Ohnishi, T.; Fujioka, S.; Watanabe, B.; Mizutani, M. Rice CYP90D2 and CYP90D3 catalyze C-23 hydroxylation of brassinosteroids in vitro. *Plant Physiol Bioch* **2012**, *58*, 220-226, doi:10.1016/j.plaphy.2012.07.011.

18. Han, J.Y.; Hwang, H.S.; Choi, S.W.; Kim, H.J.; Choi, Y.E. Cytochrome P450 CYP716A53v2 catalyzes the formation of protopanaxatriol from protopanaxadiol during ginsenoside biosynthesis in Panax ginseng. *Plant Cell Physiol* **2012**, *53*, 1535-1545, doi:10.1093/pcp/pcs106.

19. Chen, X.C.; Lu, Y.; Liu, Y.; Zhou, J.W.; Zhang, Y.F.; Gao, H.Y.; Li, D.; Gao, W. Identification of a cytochrome P450 from Tripterygium hypoglaucum (Levl.) Hutch that catalyzes polpunonic acid formation in celastrol biosynthesis. *Chin J Nat Medicines* **2022**, *20*, 691-700, doi:10.1016/S1875-5364(22)60205-X.

20. Morikawa, T.; Saga, H.; Hashizume, H.; Ohta, D. CYP710A genes encoding sterol C22-desaturase in Physcomitrella patens as molecular evidence for the evolutionary conservation of a sterol biosynthetic pathway in plants. *Planta* **2009**, *229*, 1311-1322, doi:10.1007/s00425-009-0916-4.

21. Banerjee, A.; Hamberger, B. P450s controlling metabolic bifurcations in plant terpene specialized metabolism. *Phytochem Rev* **2018**, *17*, 81-111, doi:10.1007/s11101-017-9530-4.

22. Wei, K.F.; Chen, H.Q. Global identification, structural analysis and expression characterization of cytochrome P450 monooxygenase superfamily in rice. *Bmc Genomics* **2018**, *19*, doi:ARTN 35

10.1186/s12864-017-4425-8.

23. Schmelz, E.A.; Huffaker, A.; Sims, J.W.; Christensen, S.A.; Lu, X.; Okada, K.; Peters, R.J. Biosynthesis, elicitation and roles of monocot terpenoid phytoalexins. *Plant Journal* **2014**, *79*, 659-678, doi:10.1111/tpj.12436.

24. Yamada, Y.; Motomura, Y.; Sato, F. CjbHLH1 homologs regulate sanguinarine biosynthesis in Eschscholzia californica cells. *Plant Cell Physiol* **2015**, *56*, 1019-1030, doi:10.1093/pcp/pcv027.

25. Yamada, Y.; Shimada, T.; Motomura, Y.; Sato, F. Modulation of benzylisoquinoline alkaloid biosynthesis by heterologous expression of CjWRKY1 in Eschscholzia californica cells. *Plos One* **2017**, *12*, doi:ARTN e0186953

10.1371/journal.pone.0186953.

26. Takemura, T.; Ikezawa, N.; Iwasa, K.; Sato, F. Molecular cloning and characterization of a cytochrome P450 in sanguinarine biosynthesis from Eschscholzia californica cells. *Phytochemistry* **2013**, *91*, 100-108, doi:10.1016/j.phytochem.2012.02.013.

27. Wang, W.P.; Hou, L.X.; Li, S.; Li, J.R. The Functional Characterization of DzCYP72A12-4 Related to Diosgenin Biosynthesis and Drought Adaptability in Dioscorea zingiberensis. *International Journal of Molecular Sciences* **2023**, *24*, doi:ARTN 8430

10.3390/ijms24098430.

28. Vasav, A.P.; Godbole, R.C.; Darshetkar, A.M.; Pable, A.A.; Barvkar, V.T. Functional genomics-enabled characterization of CYP81B140 and CYP81B141 from Plumbago zeylanica L. substantiates their involvement in plumbagin biosynthesis. *Planta* **2022**, *256*, doi:ARTN 102

10.1007/s00425-022-04014-x.

29. Liu, C.J.; Huhman, D.; Sumner, L.W.; Dixon, R.A. Regiospecific hydroxylation of isoflavones by cytochrome P450 81E enzymes from Medicago truncatula. *Plant Journal* **2003**, *36*, 471-484, doi:10.1046/j.1365-313X.2003.01893.x.

30. Akashi, T.; Aoki, T.; Ayabe, S. CYP81E1, a cytochrome P450 cDNA of licorice (Glycyrrhiza echinata L.), encodes isoflavone 2 '-hydroxylase. *Biochem Bioph Res Co* **1998**, *251*, 67-70, doi:DOI 10.1006/bbrc.1998.9414.

31. Overkamp, S.; Hein, F.; Barz, W. Cloning and characterization of eight cytochrome P450 cDNAs from chickpea (Cicer arietinum L.) cell suspension cultures. *Plant Sci* **2000**, *155*, 101-108, doi:Doi 10.1016/S0168-9452(00)00214-4.
